# Mathematical modeling of regulatory networks of intracellular processes – Aims and selected methods

**DOI:** 10.1016/j.csbj.2023.02.006

**Published:** 2023-02-08

**Authors:** Malgorzata Kardynska, Daria Kogut, Marcin Pacholczyk, Jaroslaw Smieja

**Affiliations:** a Dept. of Biosensors and Processing of Biomedical Signals, Silesian University of Technology, Gliwice, Poland; b Dept. of Systems Biology and Engineering, Silesian University of Technology, Gliwice, Poland

**Keywords:** Regulatory networks, Signaling pathways, Modeling

## Abstract

Regulatory networks structure and signaling pathways dynamics are uncovered in time- and resource consuming experimental work. However, it is increasingly supported by modeling, analytical and computational techniques as well as discrete mathematics and artificial intelligence applied to to extract knowledge from existing databases. This review is focused on mathematical modeling used to analyze dynamics and robustness of these networks. This paper presents a review of selected modeling methods that facilitate advances in molecular biology.

## Graphical Abstract

**Figure:**
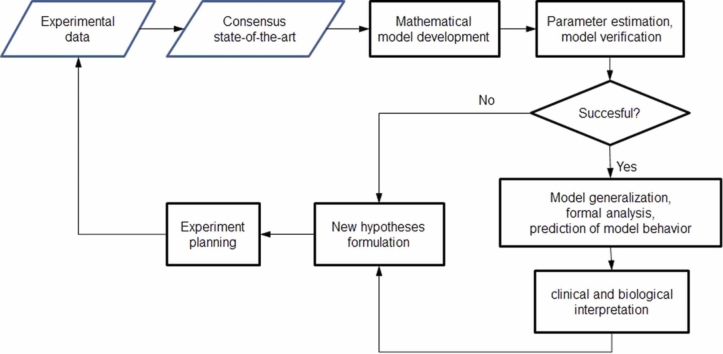

## 1 Introduction

Biological systems have been a subject of mathematical analysis for a very long time. Researchers are still working on what is the best approach to describe mathematically the behavior of such systems. From the very beginning of mathematical exploits in the fields of biology, ecology or medicine, it was clear that understanding of dynamics does not only advance knowledge of these systems, but also facilitates better management of biological resources of our planet, help to design better treatment protocols for various diseases and increase effectiveness of biotechnological industry. Models of biomedical systems describe their behavior at various levels: from populations of species to single whole-organism physiology to cell populations to intracellular processes. It is the latter that is the scope of this review.

The term “signaling pathways” relates to the cascade of processes, initiated by an external event (e.g., ligand binding to its specific receptor on a cell surface), or by an internal event (e.g., DNA damage). These processes involve creation or degradation of protein complexes, activation of enzymes and usually lead to activation or repression of transcription of genes specific for a given pathway. This results in production of new proteins (or their disappearance, if the genes are repressed) which may affect earlier stages of the cascade, thus creating positive or negative feedback loops. These loops constitute regulatory networks and their analysis is crucial to understand how cells respond to various stimuli and, ultimately, use that knowledge to develop new treatment strategies in different diseases that should be approached with precision medicine.

Following rapid developments in new experimental techniques, mathematical modeling of regulatory pathways that control intracellular biological and chemical processes has almost become a separate scientific discipline, offering large number of methods, tailored to specific purposes (see, e.g., [20], [28], [38], [68], [124] and references therein). Analysis of biological data has led to much better understanding of the nature of intracellular processes. Though our knowledge of these processes has rapidly been expanded, still much more remains to be uncovered. Research efforts are hampered by at least several factors, the large costs of experiments being not the least of them. So far, much more knowledge has been gained concerning pathways structure than their dynamics. Despite a lot of efforts, a relatively small number of models have been hitherto tested against experimental data, and, therefore, a lot of their parameters remain unknown. Moreover, due to their complexity, intertwining and lack of detailed knowledge of the mechanisms regulating each step of the signaling cascades, it is impossible to build precise mathematical description of entire pathways and take into account all factors playing a role in a realistic system. Therefore, analysis is always constrained to several most important processes. Nevertheless, the resulting models provide valuable insights into complex behavior on the cellular level [102], [123], kinetics of the involved proteins and their complexes and gives the predictions of the possible responses of whole system to the change in the level of a given activator or inhibitor or a structure of the regulatory network [130]. Thus, even simplified models can significantly contribute to the biological field.

## 2 Goals of mathematical modeling of intracellular process

Generally, the goals that can be reached by developing and analyzing mathematical models in biology and medicine can be divided into five categories:

1. Direct application in medicine, agriculture or biotechnology (e.g., [30], [143]). This can be usually achieved for the models characterized by not so many parameters that can be estimated with respectively good precision.
2. Facilitating design of synthetic biology circuits [4], [78], [106] that can further be utilized to produce desired biomolecules or biomaterials needed in biotechnology or medicine [13], [77], [127]
3. Drawing conclusions from "static" data representing features of species by means of statistical analysis (e.g., analysis of gene expression, search for biomarkers of diseases, predicting the treatment outcome, etc. (e.g., [134]). Such analysis is beyond the scope of this paper.
4. Qualitative behavior analysis - aiming at finding conditions of stability or sustained oscillations (e.g. [74], [97]). They can serve two purposes - foreseeing the fate of the system (if the model parameters can be measured),or providing clues that at least some information about the mechanisms governing system dynamics is missing (in the worst case when the parameter values are not available), because the model cannot reflect true system behavior. Very often the investigation is restricted to asymptotic analysis or analysis of stability of model stationary points. At first glance, it might seem inappropriate, as asymptotic convergence translates into reaching a particular state in infinite time. However, it provides important clues about system properties. First, it may help in explaining apparent discrepancies between experimental results obtained by different groups (as these results might have led each group to a different stationary point). It is particularly important if the processes exhibit distinct types of behavior dependent on threshold value of some variable (see, e.g., [12]). Second, by revealing the character of stationary points, it may either help to interpret experimental data or, I some cases, negatively verify a hypothesis about the interactions behind a particular process, without the need to conduct an experiment.
5. Checking if the state-of the-art knowledge about some biological system is sufficient to explain all phenomena observed experimentally. In some way, it is a subset of goals mentioned in the preceding point. However, this point is relevant particularly for models that can be treated only numerically, due to their complexity. In these cases, the emphasis is quite often put on transient states. If no parameter set can be found such that the system dynamics mirrors what is observed in experiments, it indicates that other, unknown mechanisms, or processes, are involved (e.g., [116]).

This paper is focused on the latter two categories.

Such a view on application of mathematics to describe biological systems is only one of possible approaches. It should be emphasized that finding a universal mathematical model is not the research goal due to extreme complexity of investigated systems. Usually, researchers attempt to find a mathematical description of the real world, treating it as a tool only for discovering and/or explaining biological mechanisms. It can be seen as a mathematical empirism [96], or a bottom-to-top approach (assuming that knowledge of processes at the lowest levels can be applied to simplify their description and help in understanding mechanisms regulating processes at a higher level). An alternative way would be to choose Platonic approach to mathematics, in which mathematical structure is an integral property of the world and therefore a good model reveals the truth about a system it describes [52], [55].

**Fig. 1:**
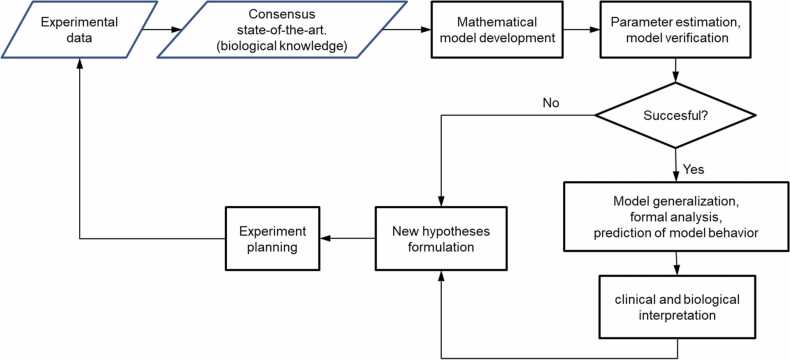
Intertwining biological and modeling research to expand knowledge in molecular biology and in biomedicine in general.

Fig. 1 illustrates how experimental work can be supported by mathematical modeling.

**Fig. 2:**
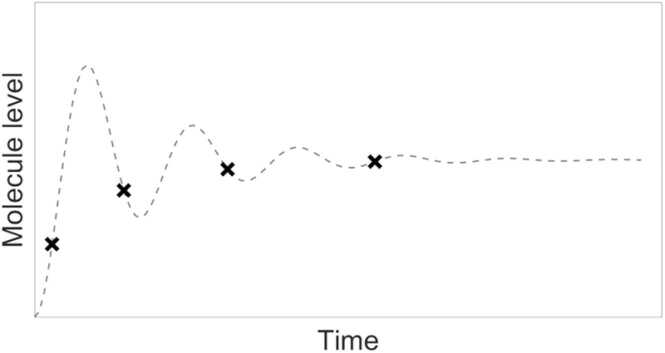
Actual time response of a signaling pathway (grey dashed line) and misleading results of experiments with several time-point measurements (black crosses).
• providing information about any system output, including one representing hypothetical molecules or molecules whose levels cannot be measured for any reason (as long, as it is represented by a variable in the model);
• facilitating faster prototyping in synthetic biology [65], thus pushing forward development of new biomaterials, biosensors and advancing medicine [22];
• indicating prospective molecular drug targets, suggested by a tailored sensitivity analysis (and the first step for subsequent molecular dynamics modeling that significantly reduces costs associated with new drugs development) (e.g., [21], [62]; see also a critical review by [111]);
• supporting therapy planning and predicting long-term response to treatment and transient (sometimes counterintuitive) changes in this response [39].

In particular, properly constructed mathematical models and their analysis may yield the following benefits (though they may be farfetched, as conclusions are based on the assumption that the model structure is correct and only feasible simplifications have been made when building it):

• answering a question if the current state-of-the-art knowledge can explain observed experimental results or if something is missing (e.g., when regardless of parameter changes, the model cannot reflect the system dynamics) – initial math model hypothesis suggests new experiments [116];
• saving resources that otherwise would be spent on experiments should not be planned (because the hypothesis to be tested will prove to false as suggested by the mathematical model) [115]
• supporting experiment planning through the number of cells that should be quantified at particular times to learn as much as possible about the model parameters and reduce the measurement error [41], or indicating, in the case of limited time-point measurements, what should be the most informative time instants to gain a proper insight into true system dynamics (Fig. 2; see also [114]; despite recent progress in live microscopy [49], [93], such measurements constitute most of laboratory experiments;

## 3 Modeling approaches and methods

Before any model of a regulatory network dynamics is built, its structure should be defined. Until recently, model development has required time-consuming literature research, unless the structure of interest existed in respective databases. That is slowly changing, as new methods for structure inference from various data have been developed, e.g. Gaussian process dynamical systems (GPDS) [90], graph neural networks [129] or sparse maximum likelihood algorithm to determine relationships that influence transcript and protein abundance [79]. An excellent review of these tools can be found in [100]. More and more often, these tools are applied not only to define regulatory network structure, but also to make viable clinical conclusions about prognostic biomarkers and molecular drug repurposing [137], [139].

Once the regulatory network or signaling pathway structure is known, its mathematical model can be developed. Though various modeling methods were employed in recent years, it seems that differential equations-based approach is the most popular. Depending on the goal of the modeling and what phenomena are to be captured (i.e., temporal, spatiotemporal, stochastic), ordinary, partial or stochastic differential equations are used. They describe either a particular biochemical system and are based on experimental data, or a generic one, to answer general questions about possible systems behavior. In such models, variables usually represent concentrations, levels or the number of molecules of molecular species that are taken into account in a given system. Sometimes, such approaches are expanded by adding a component from other methodologies, like fuzzy logic propositions [35], or combining dynamics analysis with a parallel one that is concerned with a model structure, not dynamics, as is the case with Petri nets [19], [48]. Good reviews of these methods can be found in [31], [50]. While analysis of generic systems does not give rise to controversies, models based on differential equations are criticized for having to many unidentifiable parameters, assumed arbitrarily and hence not trustworthy. The critics propose an alternative approach, in the form of Boolean networks [110] and discrete time models based on Boolean networks [44].

In general, large number of parameters that are difficult to estimate is one of the biggest obstacles in wide-scale application of the modeling approach in analysis of intracellular processes. Though parameter estimation has been the subject of research for many years (see e.g. [73], [91]), application of the methods developed is relatively straightforward only in technical or simple biological systems. Specificity of intracellular regulatory networks, consisting in intertwining of large-scale subsystems makes it very difficult to directly apply these methods in this area of research, despite many attempts to do so (e.g., [71], [117]).

It should be emphasized, that the approaches mentioned above make it possible to perform not only simulation-based, computational analysis, but also a more formal one, focused on asymptotic behavior, multiple stationary points, stability conditions, oscillation conditions, model robustness, etc. At the other end of the spectrum are agent-based models (e.g., [82]), facilitating a wide range of heterogeneous responses of individual cells, but in simulations only.

The range of specific signaling pathways and regulatory networks, whose investigation is supported by mathematical modeling seems to be unlimited. Each year, new models appear, describing systems that previously have not been dealt with. However, most efforts concentrate on pathways involved in determination of cell fate [64], cell cycle [3], response to stress [63], [89], carcinogenesis [125], immune system responses [29], [76], or, most recently, micro-RNA-mediated regulation of intracellular processes [61], [92], [104].

## 4 Linking computational intracellular models with pharmacokinetics and pharmacodynamics

Metabolic networks or signaling pathways modeling are important tools for analysis of processes at the intracellular level. While first models of metabolism at the genome scale considered only generic collection of processes in living cells, more recent approaches account for network structure in specific tissues, allowing for the consideration of cellular models within the context of whole-body physiology. Therefore, numerous mathematical modeling methodologies have been proposed to describe physiological processes at different levels of biological organization.

Signaling pathway models are well suited to the investigation of in vitro experiments with usually well-defined and equilibrated conditions but they do not suffice for meaningful analysis of in vivo metabolism, where the ever-changing environment of the surrounding tissue and organism strongly affects metabolism at the cellular level. Living cell metabolism can only be fully understood through a complete analysis while addressing the context of the whole body. Several methods have already been described to combine different levels of biological organization: cell-level pharmacokinetics (PK) and pharmacodynamics (PD) to characterize efficacy of antibody-drug conjugates [113], successful application of reconstruction of a human hepatocyte genome-scale network into the liver tissue of a physiologically based pharmacokinetic model [66] or multiscale cell-type–specific PK/PD models for personalized medicine [6].

Physiologically based pharmacokinetic (PBPK) models [67], in contrast to more general classical PK and PD models [26], offer mechanistic and quantitative representation of absorption, distribution, metabolism, and excretion (ADME) related processes of naturally occurring and exogenous molecules within living organisms at the whole-body level. The structure of the PBPK model consists of compartments representing all relevant organs, tissues, and the systemic circulation. Initially, PBPK model contains certain amount of prior physiological and anatomical information as well as distribution models (e.g., relative gene expression in particular compartment) collected from available databases or literature. Most model parameters are already integrated in the modeling software (e.g., PK-Sim/Mobi) [133] or can be easily calculated from the physiochemistry of the analyzed compound (e.g., lipophilicity, molecular mass, solubility, etc.). Initial parameter values can be further optimized using collected experimental measurements [37], [105]. Although PBPK models contain hundreds of ODEs, variables and parameters, the number of parameters that need to be optimized during model development is usually acceptably small [66]. Selected parts of PBPK models can be prepared at the greater level of detail depending on particular region of interest. There are many examples of PBPK models fine-tuned for special requirements (e.g., drugs administered by inhalation) [36]. PBPK models found their use in analysis of drug pharmacokinetics [142], pharmacogenomics [46], species extrapolation [54], and special populations (children [8], women during pregnancy [25], diabetes [72]). Number of submissions containing PBPK studies to regulatory authorities (FDA, EMA and PMDA, Japan) is rapidly increasing over last decades [34]. Drug agencies strongly encourage use of PBPK modeling at all stages of drug development especially in special populations due to safety risks involved [112].

## 5 Software tools and databases

In recent years, both in vitro and clinical research have been supported by expanding databases and computational tools developed either by standalone software, web-based applications or various toolboxes (e.g., one of the newest ones by Calzone et al. [16]). These are great tools that collect a lot of interesting information in one place, allow for a comprehensive review of existing information, and often offer the possibility of data analysis and visualization. Below, a brief review of the latest proposals for databases and toolboxes (or their updates) has been presented, with a summary given in Table 1 (in which, the most popular and widely known KEGG and Ensemble have not been included).

**Table 1:** Databases and computational resources supporting analysis of regulatory networks.

Name	Type	Characteristics	Literature
GeneMANIA	Software	Gene function predictions (e.g. identification of potential biomarkers of disease).	[85] [131]
GEPIA	Database/Software	Gene expression analysis, complex data analysis, Differential analysis, Survival analysis, Correlation analysis, Similar genes finder,	[120]
Autophagy Regulatory Network	Database	Autophagy proteins – signaling pathway connections (integrated and predicted data)	[122]
dbPepNeo	Database	Prediction of presence of neoantigenes in different types of cancers. Mining of tumor-specific antigens based on NGS.	[119]
The human protein atlas	Database	Expression data for proteins in every kind of tissue/origin/cell line etc.	[121]
UALCAN	Software	Tools for analysis of transcriptome and proteome expression, Survival analysis	[18]
GRAND	Database/Software	miRNA and GRN analysis, enrichment analysis for TFs, comparing gene expression for normal and cancer tissues, and finding potential drugs, according to results of gene expression	[11]
NetworkAnalyst 3.0	Software	Tool for visual analysis of GRN, gene expression, microarrays and signaling pathways	[140], [141]
KOBAS-i	Software	Visualizations of gene enrichment analysis	[15]
Consensus Gene Regulatory Network Construction	Web Software	Statistical analysis of GRN	[103]
GeNeCK	TWeb server	Construction and visualization of GRN	[138]
GeRNet	Software	Visualization of gene expression analysis, visualization and statistical analysis of GRN.	[33]

The main topic of interest of the teams is the creation of tools that deal with the analysis of the gene regulatory network (GRN). In recent years, dozens of tools have been developed to analyze such data. A great example of how to use the potential of these tools is the work [83], in which several existing bioinformatic tools were used for a comprehensive analysis of the characteristics of ovarian cancers. Tools were used to investigate both signaling pathways and predict interactions of selected genes [85], [131]. To obtain more comprehensive results, tools using sequencing (TCGA) and tissue databases (GTEx) were also used [120]. The tools used to assist in the interpretation of biological data can be divided into two groups: databases that collect information on a specific topic, and toolboxes that allow visualization and comprehensive data analysis. In [122], the database focuses solely on the autophagy process. All dependencies and relationships were created as a result of manually selected interactions and components taking a smaller or greater part in this process. The database proposed by Tan et al. [119], in turn, focuses on collecting data for neoantigen peptides, based on data obtained from mass spectrometry or the results of immunological tests. This database mainly contains general information on manually found neoantigens, i.e., basic biological data and basic statistical data. The database described by [121] collects human proteomic data, where a map of the entire proteome is illustrated and protein expression patterns based on immunohistochemistry and immunocytochemistry data. Of course, there are also very extensive databases that offer the user access to comprehensive and advanced analyses, e.g., KEGG [60], Panther Pathways [84] or Ensembl [27]. Some of these are meant to be more general, some are built for a specific purpose, like UALCAN, devoted to cancer data [18]. These databases often also use the resources of other databases, allowing for faster search by gene names, aliases, and various ID numbers, which are different for each database. A very interesting and valuable example of a database is the GRAND database proposed by Ben Guebila et al. [11]. It is a database that uses many toolboxes available on the Web and allows for a deeper analysis of GRNs (gene-regulatory networks). The database uses tools for miRNA analysis (PUMA), to build single-sample GRNs (LIONESS), to construct GRNs using relaxed graph matching (OTTER), and to use Gaussian graphical models to build multiomic networks (DRAGON). The data used in this database were taken from the GTEx, TCGA and GEO databases, and the multiomics data were used from the measured CMAP CCLE database and the data were used.

The second group of tools are publicly available toolboxes for data analysis. They offer a wide range of analyzes, ranging from sequencing and microarray analyses to analyses of protein relationships and transcription factors in signaling pathways. In addition, most of these tools also offer the ability to visualize data,

NetworkAnalyst 3.0 is a tool described by Zhou et al. [140], [141]. It is a tool for visualizing genomic and proteomic data for 17 different species, using several related databases. This tool allows you to create diagrams, e.g., PPI (protein-protein interaction), TF-gene interaction, protein-drug interaction, or gene-miRNA interaction, and allows one to generate heatmaps for a more transparent analysis. NetworkAnalyst also has basic tools for simple bioinformatics analysis, such as filtering, normalization DE (differentially expressed), and meta-analysis. The latest update is also enriched with the creation of the most popular plot types (volcano plot, boxplot, Venn).

The KOBAS-i tool proposed by Bu et al. [15] presents a completely different way of analysis, based on machine learning. This database combines FCS (functional class scoring) and PT (pathway topology) tools that intelligently design signaling pathways by prioritizing appropriate biological pathways. Like NetworkAnalyst, this tool uses multiple databases and allows a clear visualization of the results.

Sarkar et al. [103] developed a tool that constructs the GRN using four statistical methods: correlation, principal component regression, partial least squares, and ridge regression. Probability values are computed for edges from the mixture distribution of edge scores obtained from each method. The probability values are combined using Fisher's weighted method. The tool does not have a visualization module, but the authors provide the names of the tools for which the output files are compatible. The GeNeCK tool [138] works similarly, also uses statistical methods, but the choice of methods is different. Like the previous tool, GeNeCK does not have a data visualization module.

Another way to analyze signaling pathways is to analyze on microarray data. Dussaut et al. [33] proposed the GeRNet tool, which is based on the GRNCOP2 algorithm [43], which enables the construction of a signal path, and BiHEA [42], which is a biclustering algorithm that finds coexpression between genes.

One of the most important criteria for tools is the user-friendly interface. This is important because such databases are used mainly by biologists who have a basic background in bioinformatics. One of the most clearly created interfaces is the GRAND database [11], where both the database and the description in the article are comprehensive and transparent. Furthermore, this tool contains very extensive resources and a clear visualization of the results.

## 6 Success stories

One of the main goals of mathematically modeling signaling pathways and regulatory networks, mentioned in the previous sections is to provide hypotheses about missing components in current knowledge about their structure and dynamics. One of the examples of such work can be found in [116], where the authors suggested that in order to explain experimental results activation of some phosphatase, not yet identified, is needed. That was confirmed much later [14], and that finding, in turn, allowed to develop a new model explaining JAK-STAT network's ability to decode relative changes of dose, timing, and type of temporal interferon stimulation [59].

On the other hand, while mathematical modeling provides convenient computational mechanisms to test biological hypotheses about unknown regulatory mechanisms, its results should be treated with caution and not regarded as the ultimate proof. Concerning the size and complexity of the models, in most cases one might propose alternative mechanisms and explanations and fit the resulting model to experimental data showing the relevance of the alternative. It is biological experiments that provide the ultimate proof for any hypothesis. Nevertheless, the modeling provides rationale for discarding some of the alternative explanations when the model structure does not allow to fit experimental data, regardless of parameter values (see e.g. [63] for computational analysis of possible mechanisms behind the heat shock and NF-κB pathways), thus saving resources needed for experiments.

Mathematical modeling has a long and successful history in assisting the optimization of therapeutic protocols. Integrating pharmacokinetics and pharmacodynamics modeling into a model describing a particular process could help to determine the best dosing strategy for best efficacy while maintaining toxicity constraints [7]. This can be achieved, for example, by minimizing an appropriately defined objective function which corresponds to a classic constrained nonlinear optimization problem [70], [118]. In the case of anticancer therapies this can be described e.g., by estimating the optimal drug doses and the administration protocol (times of drug administration), that will lead to the minimal average tumor size over time while satisfying the hematological constraints [69]. Such a modeling strategy was first tested in MODEL1 trial, a model-driven phase I/II dose-escalation study of densified docetaxel plus epirubicin administration in metastatic breast cancer patients [53], [135]. Such a therapeutic protocol has previously been studied without the use of mathematical modeling support, however often had to be discontinued because of life-threatening or lethal hematological toxicities [23], [101]. The model-driven dosage adjustment allowed for better efficacy-toxicity balance in patients with cancer when several anticancer drugs are combined [53].

Mathematical modeling can also assist in the interpretation of the results of clinical trials. ODE-based models are irreplaceable tools to capture non-linearity in biomedical data. The non-linearity included in a model captures the dynamics of the system and can yield counterintuitive and useful predictions. A good example is the modeling of tyrosine kinase inhibitors (TKIs) therapy in patients with chronic myeloid leukemia (CML) [39]. TKIs inhibit the BCR/ABL oncoprotein driving the growth and persistence of CML and thus lead to the CML remission, however, it is a standard practice to administer TKIs indefinitely because of concern about relapse [107]. Due to the high cost of therapy and the side effects associated with it, the possibility of a safe dose reduction or discontinuation of therapy without the risk of relapse is being investigated [75], [87], [98]. Based on data from clinical trial Fassoni et al. [39] developed a mathematical model and proposed a safe strategy of dose reduction of TKIs for CML patients in chronic phase. According to their model, halving the dose of the TKI will maintain the current level of response for most patients, however, it may cause a temporary increase in BCR/ABL transcript levels which return to baseline after continued treatment with the reduced dose. Therefore, such transitory increase in BCR/ABL levels should not be immediately classify as treatment failures and increases in BCR-ABL ratio could be permitted for up to a year, at which point second phase decay would be expected to occur [39]. Such conclusions seem counterintuitive and it is easy to imagine that many clinical trials, not only related to CML, have been considered a failure due to a temporary increase in specific transcript or protein levels. The model shows that this type of error can be avoided with the use of mathematical modeling which allow the prediction of treatment responses a priori.

Mathematical modeling can be used not only in therapy optimization, but also in drug design. A good example of the use of mathematical modeling and sensitivity analysis in drug development and a fruitful cooperation between modelers and experimentalists can be studies of the ErbB/PI3K signaling pathway aimed at the development of anticancer drugs [108]. Considering its role in therapeutic resistance the ErbB3/PI3K/Akt pathway is suggested as a major cause of treatment failure in cancer therapy [2]. Schoeberl et al. [108] developed a mathematical model and calibrated it with quantitative experimental data describing the ErbB/PI3K signaling pathway. Its sensitivity analysis suggested inhibition of ligand binding to ErbB3 (also known as HER3) as a more successful approach in cancers associated with ligand-induced stimulation of ErbB-PI3K signaling mediated by combinatorial receptor activation. Furthermore, they developed MM-121 (later named seribantumab), a fully human anti-ErbB3 (anti-HER3) monoclonal antibody that induces ErbB3 internalization and degradation [109]. Seribantumab has been investigated in numerous clinical trials (phase I and II) in combination with anti-cancer drugs or chemotherapy that covers a broad spectrum of cancer patients ([24], [32], [56], and many others) and research on it is still ongoing.

Over time, more sophisticated methods have been developed to aid the drug development process. A recent work presents a variance-based sensitivity analysis method that has been tailored to the search for drug molecular targets [62]. The proposed method allows studying the drug-induced changes in model responses resulting from the action of hypothetical drugs that successively target the processes described in the model and at the same time tackle the problem of heterogeneity of cellular responses to a drug in a cell population [40], [88]. The method was used, inter alia, to analyze the p53 signaling pathway whose dysregulation is observed in many cancer types [1]. The analysis was focused on finding drugs inducing a high level of p53 in cells and ultimately leading to their death [88]. The conducted analysis showed a high potential for therapies that would target Mdm2 mRNA, which can be done e.g., through devising siRNA as proposed in another *in silico* study [95] and is generally in line with the latest trends in RNA drug research [99], [136]. Among others, the analysis also showed a significant therapeutic effect for the drugs disrupting Mdm2-induced degradation of p53. In fact, such a drug has already been clinically tested [126] and similar compounds are still being investigated in anti-cancer research [9], [10].

Mathematical modeling combined with synthetic biology has raised increasing interest recently. Their application to development of novel treatment strategies, targeting components of signaling pathways that either have been damaged by a disease or, as in cancer, are the reason for failing regulatory networks, opens a completely new field in medicine. Rewiring of Aberrant Signaling to Effector Release (RASER) has been reported to specifically detect an intracellular oncogenic state and change the failed signaling pathway to trigger therapeutic effects [22]. The applicability of this system has been proven using in vitro experiments with cancer cell lines. It should be emphasized that its development was preceded by a thorough analysis of a mathematical model in the form of ordinary differential equations Such modeling approach was also used in development of an optogenetic system for the rapid down-regulation of protein levels [5] and production of biomaterials [128].

A good review of cell-free synthetic biology platforms, including with mathematical modeling that supports them, can be found in ([58], [86]). The work by Mauri et al., [80] presents an interesting discussion of application of synthetic biology in biotechnology sector and provides a mathematical modeling framework that can be used to analyze possible outcomes resulting from it.

## 7 Examples of the use of Petri nets

The literature also provides examples of the successful use of Petri nets used to search for molecular targets for drugs. Similarly, to the ODE-based models, the analysis of the Petri nets allows the identification of key elements in the regulatory network. These key elements can become molecular targets for drugs used to either restore normal signaling in cells (e.g., in diseases associated with disorders of this signaling) or, on the contrary, to interrupt signaling in a certain group of organisms (e.g., bacteria). In recent research, the bioenergetics of Mycobacterium tuberculosis (Mtb) were studied using Petri net [47]. Mycobacterium, for its growth and pathogenicity, depends on the oxidative phosphorylation pathway which is unique in Mtb due to the differences in complexes carrying electron transfer [57]. The proposed Petri net explains the processes occurring within the electron transport chain of Mtb. Using modeling and invariant analysis techniques authors investigated the impact of uncouplers, molecules that inhibit adenosine triphosphate (ATP) synthesis by uncoupling oxidative phosphorylation from the electron transport chain on Mtb. The analysis revealed that in the presence of uncouplers, there is no ATP synthesis which leads to the death of Mtb cells. The development of uncouplers to be used as an anti-mycobacterial drug is still a research area, but the potential of uncouplers as medicine is being explored in nowadays research [45], [51], [57].

Another example is research on β-thalassemia, sickle-cell anemia, and other β-globin disorders caused by mutations in adult hemoglobin (HbA), very common genetic diseases affecting millions of patients worldwide. Since it has been shown that an increase in fetal hemoglobin (HbF) levels relieves the clinical severity and decreases mortality in sickle-cell anemia and β-thalassemia [17], [94], [132] research is carried out on an effective way to raise its level. A hybrid functional Petri net (HFPN) has been used to model the human fetal-to-adult hemoglobin switch network and perform a comparative analysis of treatment with available drugs (ST-20), drugs in clinical trials (Simvastatin, tBHQ, MS-275, and ACY-957), as well as hypothetical drug inhibiting Erythroid Transcription Factors (ETF) proposed by authors, which turned out to be most efficient drug target among the tested [81]. Simulation results have been verified with qPCR data available for known drugs.

Surprisingly, although various modeling approaches have been used to detect key elements in disease-related signaling pathways and have led to numerous promising molecular targets for drugs, recent work shows that the results obtained with different methods do not always overlap [48]. Comprehensive structural and parametric analysis of ATM/p53/NF-κB signaling pathway model based on ODEs and Petri net has been performed and resulted in finding different subsets of important processes that might indicate prospective targets for drugs. While some of the processes appeared in each analysis method, others were found only by one of them and would be missed if only that method was applied [48]. This discovery emphasizes that each method has its own advantages and limitations, and none is better than the other. Therefore, the creation of multidisciplinary teams that can analyze the problem from different angles and use various methods and tools should be a standard in conducting modern research.

## 8 Concluding remarks

Mathematical modeling of signaling pathways and regulatory networks provides strong support for research in molecular biology and medicine. At present, the models are mostly used to confirm or reject some hypotheses about biochemical regulatory processes that take place inside cells, or explain some phenomena observed in experiments. In particular, possibility of rejection of some hypotheses is valuable, as it helps saving resources on unnecessary experiments. One should remember, however, that model-based confirmation of a hypothesis is only the first step, since it can be accepted only after its experimental confirmation. But even now, modeling facilitates new drug development, in particular in combination with molecular dynamics modeling. It may ultimately lead to other possible direct applications in clinics, e.g. in terms of supporting development of therapy protocols. More an more models that describe intracellular processes, should eventually lead to better understanding on cell populations behavior and, ultimately to development of multiscale models that will be much closer to clinical applications.

It should be noted that, especially concerning signaling pathways and cancer modeling, there are various approaches and each time the one that is chosen should be tailored to a specific task to be performed. There are a lot of open questions, concerning e.g. intercellular signaling among cancer cells, influence of so called crowding effect on signaling pathways and many others. Perhaps the most interesting is how stochastic nature of the intracellular processes leads to stable and quite predictable behavior of the cell as a whole system (not to mention cell populations and tissues). Though some mathematical work has been done in this field, they are far from being completed.

Finally, it should be emphasized that the models should be created to answer particular questions arising in investigation of biological systems and not only as a mathematical challenge.

## CRediT authorship contribution statement

**Malgorzata Kardynska:** Investigation, Writing – original draft, Writing – review & editing, Funding acquisition. **Daria Kogut**: Investigation, Data curation, Writing – original draft, Writing – review & editing, Funding acquisition. **Marcin Pacholczyk**: Investigation, Writing – original draft, Writing – review & editing, Funding acquisition. **Jaroslaw Smieja**: Conceptualization, Investigation, Writing – original draft, Writing – review & editing, Visualization, Supervision, Project administration, Funding acquisition.
